# Migrating Mule Deer: Effects of Anthropogenically Altered Landscapes

**DOI:** 10.1371/journal.pone.0064548

**Published:** 2013-05-14

**Authors:** Patrick E. Lendrum, Charles R. Anderson, Kevin L. Monteith, Jonathan A. Jenks, R. Terry Bowyer

**Affiliations:** 1 Department of Biological Sciences, Idaho State University, Pocatello, Idaho, United States of America; 2 Colorado Division of Parks and Wildlife, Grand Junction, Colorado, United States of America; 3 Wyoming Cooperative Fish and Wildlife Research Unit, University of Wyoming, Laramie, Wyoming, United States of America; 4 Department of Natural Resource Management, South Dakota State University, Brookings, South Dakota, United States of America; Ben-Gurion University of the Negev, Israel

## Abstract

**Background:**

Migration is an adaptive strategy that enables animals to enhance resource availability and reduce risk of predation at a broad geographic scale. Ungulate migrations generally occur along traditional routes, many of which have been disrupted by anthropogenic disturbances. Spring migration in ungulates is of particular importance for conservation planning, because it is closely coupled with timing of parturition. The degree to which oil and gas development affects migratory patterns, and whether ungulate migration is sufficiently plastic to compensate for such changes, warrants additional study to better understand this critical conservation issue.

**Methodology/Principal Findings:**

We studied timing and synchrony of departure from winter range and arrival to summer range of female mule deer (*Odocoileus hemionus*) in northwestern Colorado, USA, which has one of the largest natural-gas reserves currently under development in North America. We hypothesized that in addition to local weather, plant phenology, and individual life-history characteristics, patterns of spring migration would be modified by disturbances associated with natural-gas extraction. We captured 205 adult female mule deer, equipped them with GPS collars, and observed patterns of spring migration during 2008–2010.

**Conclusions/Significance:**

Timing of spring migration was related to winter weather (particularly snow depth) and access to emerging vegetation, which varied among years, but was highly synchronous across study areas within years. Additionally, timing of migration was influenced by the collective effects of anthropogenic disturbance, rate of travel, distance traveled, and body condition of adult females. Rates of travel were more rapid over shorter migration distances in areas of high natural-gas development resulting in the delayed departure, but early arrival for females migrating in areas with high development compared with less-developed areas. Such shifts in behavior could have consequences for timing of arrival on birthing areas, especially where mule deer migrate over longer distances or for greater durations.

## Introduction

Ungulates generally migrate along traditional routes [1], [2], and demonstrate high fidelity to seasonal ranges [3]. Recently, however, many of those migratory routes have been disrupted by increasing levels of anthropogenic disturbance [4], [5]. Threats to remaining long-distance migration of ungulates include energy development, tourism, urban sprawl, highway mortality, and habitat fragmentation [6]. If traditional migration routes are blocked or impeded, individuals may not be able to modify their migratory behavior, which could compromise persistence of those populations [2]. How overlap of migration routes with oil and gas development will affect migratory patterns, and whether ungulate migration is sufficiently plastic to compensate for such change is uncertain. Understanding effects of those disturbances on the ability of migratory ungulates to follow phenological gradients and thereby maximize energy intake, especially when parturition is looming, is of particular importance for conservation planning [7]. Indeed, the demise of migrating populations of large mammals is of increasing concern as extraction of non-renewable resources proceeds at unprecedented rates [4], [6].

Migration is an adaptive strategy that is thought to allow animals to minimize resource shortages and reduce risk of predation [8] at broad geographic scales [9], both of which affect fitness [10]. In temperate and arctic environments, heterogeneous forages may be difficult to exploit by remaining sedentary, because forage quantity and quality are seasonally and spatially dynamic [3]. Migration thereby provides nutritional advantages to individuals that exploit seasonal peaks in resources at different locations, while avoiding inclement weather and reducing intraspecific competition [11], [12].

Across western North America, mule deer (*Odocoileus hemionus*), North American elk (*Cervus elaphus*), pronghorn (*Antilocapra americana*), moose (*Alces alces*), and bighorn sheep (*Ovis canadensis*) may migrate 50–260 km between seasonal ranges [2]. Ungulate migrations in temperate environments are typified by movements between high elevations in summer and low elevations in winter [3], [12], [13]. Winter ranges are typically smaller and populations occur at relatively high density; therefore, movement to spring and summer ranges provides a release from a restricted food supply during a time when costs of reproduction are rising [8], [12], [14].

We evaluated factors that influenced patterns of spring migration for mule deer in the Piceance Basin in northwestern Colorado, USA, which included areas that were exposed to a wide range of natural-gas development. The deer in this study were the same population studied to examine resource selection along migration routes in Lendrum et al. [15]. Here we strive to determine how extrinsic and intrinsic factors influenced the timing of migration – we used different inferential techniques and a different focus, as well as a separate suite of hypotheses. Based on previous research [3], [12], we expected timing of spring migration to be influenced by extrinsic factors associated with local weather and plant phenology. Onset of spring migration often is initiated by rising temperatures, decreasing snow cover, and increasing plant growth [3], [12]. Accordingly, we tested whether spring migrations would be initiated by some combination of increased solar radiation and temperature, decreased snow depth, and advancing plant phenology. In addition, we predicted that deer residing at high elevations during summer, where snow depths were likely greater and green-up delayed [11], [16], would either postpone initiation of, or exhibit a slower rate of migration than deer inhabiting lower-elevation ranges during summer.

Although patterns of migration may be synchronized by environmental factors, migratory decisions can vary among individuals depending upon life-history characteristics and nutritional condition [12], [17]. For example, Monteith et al. [12] observed that old female mule deer in the Sierra Nevada risked encountering severe weather by delaying autumn migration. Assessment of the relative vulnerability of migratory populations requires careful consideration of both extrinsic and intrinsic factors. Consequently, we predicted that older, more-experienced deer, and those in comparatively good condition would initiate spring migration earlier compared with young, inexperienced deer, and those in relatively poor condition [3], [12].

Disturbances have the potential to override the contribution of other factors to ungulate migrations [5], [7]. After accounting for effects of environmental and individual-based factors on patterns of migration, we further hypothesized that migration would be modified by the presence of natural-gas development. Lendrum et al. [15] documented longer step lengths by mule deer migrating through areas of greater development; however they did not examine what effects this pattern of movement may have had on overall timing of migration. Similarly, Sawyer et al. [18] observed that deer migrated faster through areas with a higher density of development compared with when the same deer were migrating through less-developed areas. Because mule deer sometimes avoid oil and gas developments [19], [20], we predicted an early departure, faster movement rate, and early arrival for individuals experiencing areas with higher levels of natural-gas extraction, compared with areas of less development.

## Methods

### Ethics Statement

All aspects of animal handling and research complied with the methods adopted by the American Society of Mammalogists [21], and were approved by an Animal Care and Use Committee at Idaho State University (protocol # 670 0410). Permission to conduct all aspects of this field study was provided, in a collaborative effort, by Colorado Division of Parks and Wildlife and the U.S. Bureau of Land Management.

### Study Area

The Piceance Basin supports one of the largest populations of migratory mule deer in North America, historically estimated at 21,000–27,000 animals [22]. This area also includes one of the largest natural-gas reserves in North America. Energy development throughout northwestern Colorado is projected to increase from approximately 500 to 17,000 wells over the next 20 years (U.S. Bureau of Land Management Executive Summary 2007).

The Piceance Basin is topographically diverse and characterized by pinyon pine (*Pinus edulis*)-Utah juniper (*Juniperus osteosperma*) shrubland. Climate was typified by warm, dry summers (28°C high average) and cold winters (−12°C low average), with most annual moisture coming from snow melt in spring (Western Regional Climate Center, 1893–2010). This area was dissected by numerous drainages with stands of big sagebrush (*Artemisia tridentate*), saltbrush (*Atriplex* spp.), black greasewood (*Sarcobatus vermiculatus*), and rabbitbrush (*Crysothamnus* spp.), with most of the primary drainage bottoms converted to fields of mixed-grass hay. Primary winter habitat for mule deer ranged from 1,675 to 2,285 m in elevation. Summer range for mule deer occurred at high elevations (2,100 to 2,700 m) with dominant vegetation communities of quaking aspen (*Populus tremuloides*)-Douglas-fir (*Pseudotsuga menziesii*) and Engelmann spruce (*Picea engelmannii*)-subalpine fir (*Abies lasiocarpa*) forests [3]. The area contained additional large herbivores including North American elk (*Cervus elaphus*), wild horses (*Equus caballus*), and moose (*Alces alces*). Common species of predators included coyotes (*Canis latrans*), mountain lions (*Puma concolor*), bobcats (*Lynx rufus*), and black bears (*Ursus americana*). Lendrum et al. [15] provides a more complete description of the study area.

**Figure 1:**
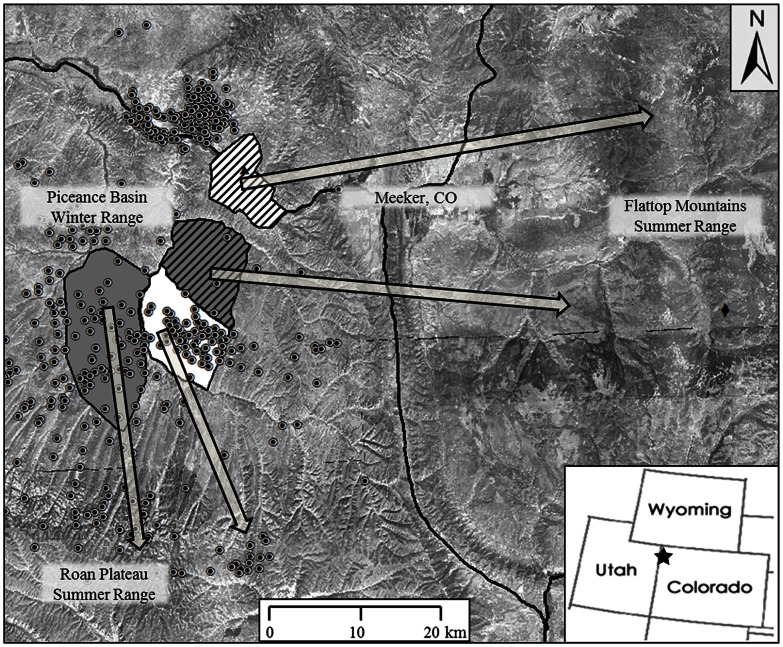
Map of study area in the Piceance Basin, Colorado, USA. Seasonal ranges and general spring migration paths (arrows) of adult female mule deer for North Ridge (white with hash marks), North Magnolia (grey with hash marks), South Magnolia (white), and Ryan Gulch (grey). Active well pads (•), Western Regional Climate Center (▴) and SNOTEL weather station (♦).

We monitored four populations of mule deer that wintered in the Piceance Basin: 1) North Ridge (53 km^2^) in the northeastern portion of the Basin; 2) Ryan Gulch (141 km^2^) in the southwestern portion of the Basin; and 3) North Magnolia (79 km^2^); and 4) South Magnolia (83 km^2^) in the central portion of the Basin (Figure 1). During spring migration, mule deer from North Ridge and North Magnolia moved easterly to higher elevations across US Highway 13 towards the Flat Top Mountain Range. Mule deer from Ryan Gulch and South Magnolia migrated southerly through a fragmented landscape of well pads, compressor stations, pipelines, and roads to higher elevations along the Roan Plateau (Figure 1). Habitat characteristics (i.e, vegetation type) occurring along the migratory paths of mule deer was similar among study areas [15], which allowed us to control for such variation while examining what other variables may have influenced patterns of migration.

### Animal Capture

We captured mule deer from a helicopter using net guns [23] to acquire a sample of adult (>1 years old) females (*n* = 205 deer; North Ridge  = 60, North Magnolia  = 43, South Magnolia  = 42, and Ryan Gulch  = 60) from 2008 to 2010. During 10–12 January 2008, we captured 45 adult female mule deer and fit them with Global Positioning Satellite (GPS) collars (14 with GPS-4400S; Lotek Wireless, Newmarket, Ontario, Canada; 31 with G2110D; Advanced Telemetry Systems, Isanti, Minnesota, USA). During late February – early March 2009 and 2010, we captured and radiocollared an additional 60 and 100 adult females, respectively (G2110D GPS collar). We programmed collars to obtain a locational fix every 5 h during the migration period, and only retained 3D fixes or 2D fixes with a positional dilution of precision <10 [24]. All collars included mortality sensors and timed drop-off mechanisms that were scheduled to release during April of the year following deployment. We retrieved GPS collars, which also were equipped with Very High Frequency (VHF) transmitters, from the field as they became available following mortalities or collar drop each spring. Of the 205 adult females collared, we censored 16 animals from analyses because they did not provide complete data on migrations.

For each adult female captured during 2009 and 2010 (*n* = 160), we measured maximum thickness (cm) of subcutaneous fat deposition at the thickest point cranial to the cranial process of the tuber ischium with a portable ultrasound device (SONOVET 2000, Canmedical, Yanker, Ontario) and a 5-MHz transducer [25]. In addition, we used palpation to determine a body-condition score, validated for mule deer, to provide an estimate of ingesta-free body fat (IFBFat), when subcutaneous fat reserves had been mobilized [26]. We used a combination of subcutaneous rump-fat thickness and body-condition score to estimate percent IFBFat [26]. We estimated deer age using tooth replacement and wear [27].

### Characteristics of Spring Migration

We used Hawth's Analysis Tools in ArcGIS 9.3 (ESRI, Redlands, California, USA) to derive 95% kernel-density estimates of seasonal ranges for each individual. We determined the initiation of spring migration based on the day a particular deer left winter range on a trajectory path (i.e., three successive locations leading away from winter range); arrival on summer range was determined as the first location inside the summer home range for that same deer [3]. We then calculated the distance and rate of travel (distance/days to complete migration) between winter and summer range along the migratory path using the Distance Between Points Tool in Hawth's Analysis Tools. We also calculated elevation of summer range for each deer as the average elevation of all locations within their summer range.

Levels of natural-gas development varied markedly among study areas within the Piceance Basin. Consequently, we calculated well-pad densities along spring migration routes for each radio-collared female. To define the width of migration corridors, we calculated the tortuosity of each movement path as log(N)/(log(N) + log(D/L)), where N is the number of line segments that make up the line, D is the distance between the start and end points of the line, and L is the cumulative length of all line segments. Tortuosity is a useful metric in linking fine-scale movements to habitat quality and human development [28]. We then used the number of well-pads within each buffered migratory path as a measure of well-pad density for individual deer. We tested for differences in characteristics among study areas and years using analysis of variance (ANOVA) with Bonferroni pairwise comparisons in Minitab 16.1.0 (State College, Pennsylvania, USA, 2010). Prior to interpretation of results, we examined residual plots of each dependent variable to test for compliance with assumptions of ANOVA.

We hypothesized that characteristics of a migratory path would influence timing of migration including: elevation at summer range (m); distance migrated (km); well-pad density (wells/km^2^); and rate of travel to summer range (km/day). Because these intrinsic variables were interrelated, we used principal components analysis (PCA), based on the correlation matrix, to reduce dimensionality of those variables and derive independent composites that described characteristics of migratory routes for individual deer (Appendix S1). Principle component 1 (PC1) explained 43.8% of the variation of the intrinsic variables, and contrasted individuals migrating quickly through high well-pad densities for a short distance (positive loadings), with those traveling longer distances at a slower rate with no well pads (negative loadings). Principle component 2 (PC2) explained 26.8% of the variation in intrinsic variables, and was associated positively with elevation on summer range.

### Local Weather

We obtained data on daily weather including: minimum, maximum, and average temperature (°C); relative humidity (%); solar radiation (Watts/m^2^); and precipitation (cm) from a weather station located within winter range on the North Ridge study area (Western Regional Climate Center 2008–2010; Figure 1). Additionally, we obtained data on snow depth from a SNOTEL weather station located near the summer range of the North Magnolia subpopulation (2,865 m; Figure 1), which served as an index to snow depth for the entire study area. In addition to absolute measures of daily weather, we calculated a metric of change in weather based on the difference in the daily weather variables relative to the mean of that particular weather variable during the previous 2 weeks, because deer may respond to changes in weather rather than to absolute values [12], [29]. We tested for effects of year on average temperature, humidity, solar radiation, and snow depth using multivariate analysis of variance (MANOVA). Following a significant main effect, we used canonical correlations to identify variables responsible for overall significance, and followed this with tests using separate ANOVAs for those variables.

We again used PCA, based on the correlation matrix, to derive independent composites that described daily weather [12]. The PCA included 14 variables representing absolute daily weather and a metric of change in daily weather for minimum, maximum, and average temperature (°C), relative humidity (%), solar radiation (Watts/m^2^), precipitation (cm), and snow depth (cm). If weather data were unavailable for a particular day (<1% were missing), we replaced the missing value with the average value of the previous and subsequent day. We selected five principal components that were both biologically relevant and explained >5% of the variation in daily weather [12]. Principle component 1 (WPC1) explained 47.3% of the variation in daily weather, and represented daily changes in temperature with an influence of humidity from cooling temperatures and moister days (negative loadings) to warming temperatures and dryer days (positive loadings). Principle component 2 (WPC2) explained 17.7% of the variation and reflected an absolute measure of daily snow depth with an influence of daily temperature from lower snow depths and warmer days (negative loadings) to higher snow depths and cooler days (positive loadings). Principle component 3 (WPC3) explained 9.9% of the variation and reflected daily changes in precipitation from wetter (negative loadings) to dryer (positive loadings) days. Principle component 4 (WPC4) explained 8.0% of the variation and was largely related to solar radiation from overcast (negative loadings) to sunny (positive loadings) days. Principle component 5 (WPC5) explained 6.5% of the variation and was almost entirely related to changes in snow depth from decreasing (negative loadings) to increasing (positive loadings) depths.

### Plant Phenology

We used the Normalized Difference Vegetation Index (NDVI) to reflect primary productivity and greenness of vegetation [30], [31], and thus, potential fluctuations in dietary quality and availability associated with spring green-up [12]. We obtained 7-day composites of NDVI from MODIS (moderate-resolution imaging spectroradiometry; ftp://emodisftp.cr.usgs.gov/eMODIS/CONUS/historical/TERRA/) with a 250-m^2^ spatial resolution. We extracted NDVI values associated with GPS locations of deer for each weekly composite. Once an individual departed winter range, we used the locations for that individual during the last week present on winter range to estimate phenological patterns for winter range for the remainder of the monitoring interval. Conversely, we used locations from the first week on summer range to extract NDVI values representative of phenological patterns on summer range, prior to arrival on summer range for each individual. We used this approach to obtain NDVI data, because simply following the path of an individual once it departed a seasonal range would inherently result in shifts in NDVI that would be caused by movement and be correlated with timing of migration, rather than allowing the assessment of the response of an individual to natural changes in greenness on a specific seasonal range.

### Modeling of Migration

We implemented time-to-event models in Program MARK to predict patterns of spring migration for mule deer, including departure from winter range and arrival on summer range. These methods were developed originally to estimate survival of marked animals [32], but they are a valuable tool for assessing factors that influence time to a specific event, and allow the incorporation of individual-based covariates [12], [33]. We estimated daily probability of not migrating as a function of extrinsic and intrinsic factors using the known-fate option in Program MARK [32], and correspondingly calculated daily probability of migrating as 1 minus the daily probability of not migrating. Beginning on 1 April, we used an 80-day interval to construct encounter histories for migration timing of each individual deer. We designated 1 April as the beginning of the interval that deer were available to migrate during each season, because that date was prior to any individual departing from winter range and the 80-day period encompassed the entire migratory period for mule deer in the Piceance Basin.

We used a two-stage process to evaluate extrinsic and intrinsic factors that were related to patterns of migration, (sensu [12], [34]). We examined extrinsic and intrinsic explanatory variables in a 2-step process because we were interested in evaluating the effects environmental variables would have on patterns of migration at the population level, and then how those patterns might be modified by intrinsic variables, which were more specific to each individual. Our first step included examining all possible combinations of extrinsic variables that we predicted would influence timing of spring migration: daily weather variables and weather-change metrics from the PCA; weekly NDVI values on both winter and summer range; year; and study area. We included year and study area as grouping variables to account for variation that was not specifically addressed by our other environmental variables.

To identify extrinsic variables that influenced timing of spring migration, we calculated Akaike's Information Criterion adjusted for small sample size (AIC*_c_*, ΔAIC*_c_*, and Akaike weight w_i_; [35]) for each model. We determined model-averaged parameter estimates and unconditional standard errors (SE) for each predictor variable [35], and evaluated the significance of each variable by whether the corresponding 90% CI overlapped 0. We also calculated importance weights as the sum of w_i_ for all models that contained a particular variable, and evaluated the relative importance of each variable based on those weights [12], [35]. We considered variables to be biologically influential if they had an importance weight of >0.70.

After identifying the extrinsic variables that influenced timing of migration, we evaluated the influence of intrinsic factors and life-history characteristics of individual mule deer. We combined the extrinsic factors identified as being influential in the first stage of the analysis with intrinsic covariates, PC1 (well-pad density, rate, and distance) and PC2 (elevation). We modeled all possible combinations of extrinsic variables that were previously determined to be important with the addition of the intrinsic variables. We again used model averaging, 90% CI, and importance weights to evaluate effects of individual characteristics on timing of spring migration [35]. In a final analysis using the same modeling approach, we evaluated the influence of age and nutritional condition of individual females, which were only collected during 2009 and 2010.

In each stage of the analysis, we also investigated effects of interaction terms among extrinsic and intrinsic variables that we hypothesized would influence patterns of migration by examining ΔAIC*_c_* and confidence intervals. We retained interaction terms in a model set if their inclusion resulted in an improvement of model fit (ΔAIC*_c_* >2.0), and their parameter estimates differed from zero [35].

## Results

**Figure 2:**
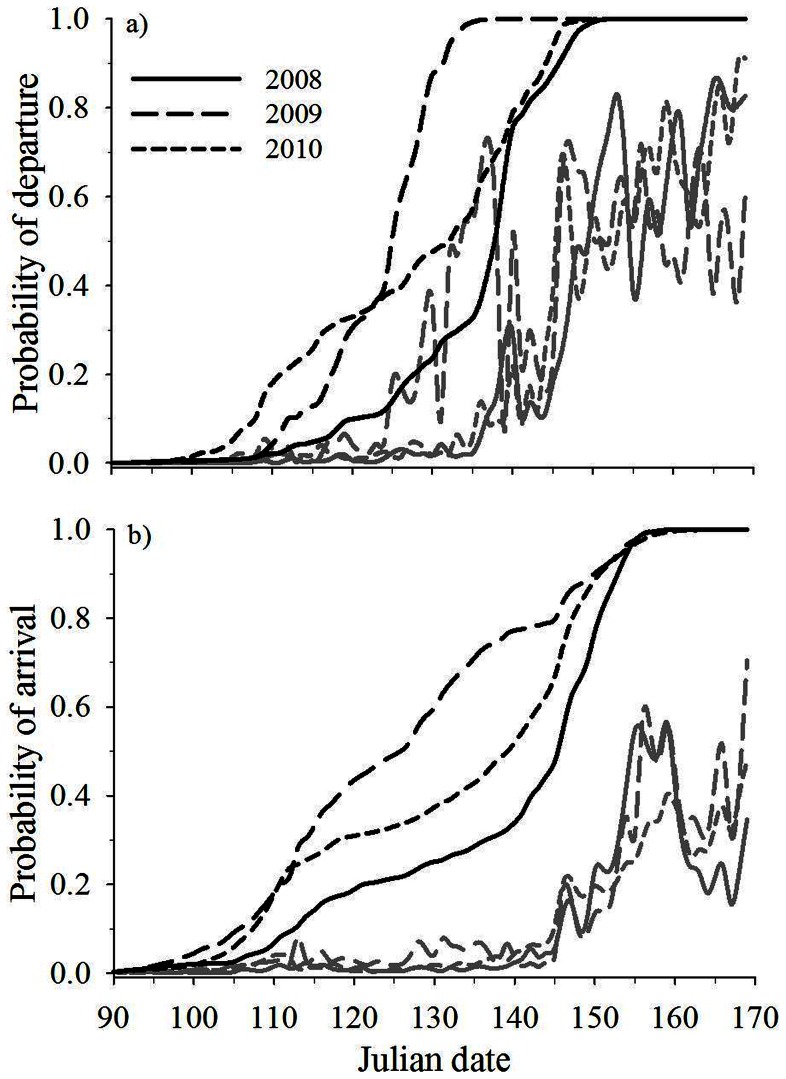
Yearly patterns of spring migration by mule deer. Model-averaged estimates of the cumulative proportion migrated (black) and the daily probability of migration (grey) relative to Julian date of departure from winter range (a) and arrival to summer range (b), for spring migration of adult female mule deer, Piceance Basin, Colorado, USA, 2008–2010.

**Figure 3:**
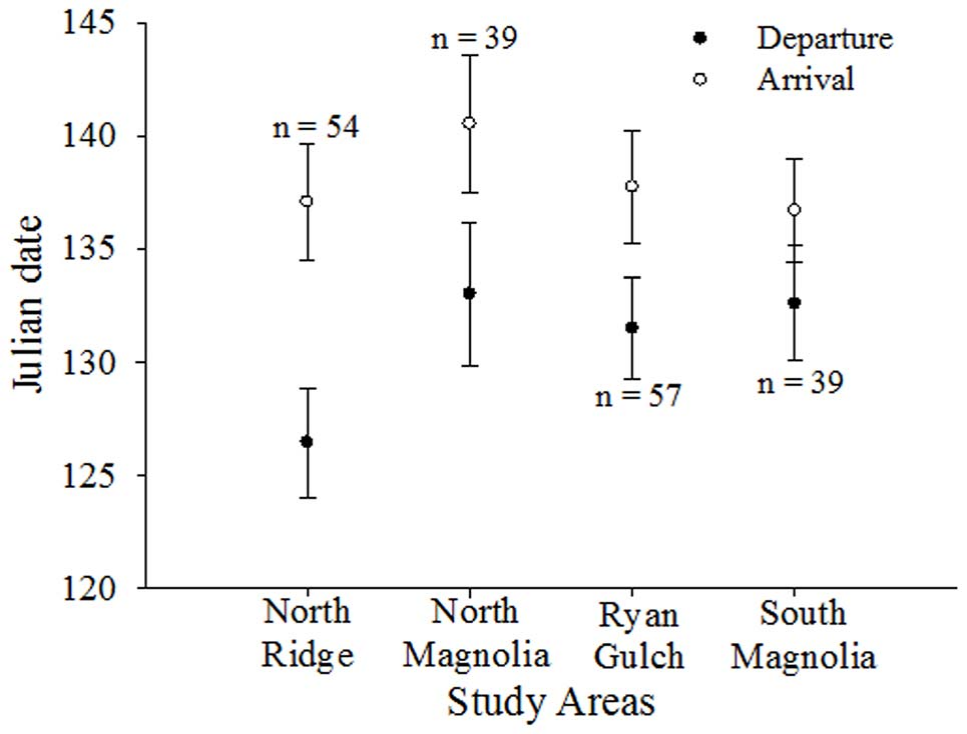
Departure and arrival dates of spring migration by mule deer. Mean Julian date (±95% CI) of departure from winter range and arrival to summer range for spring migration of adult female mule deer, Piceance Basin, Colorado, USA, 2008–2010. Mean values for study areas are of combined years and values above or below means are number of collared deer during each year.

During 2008–2010, we recorded 189 spring migrations by adult female mule deer. Mean date of departure (*F*_38,188_  = 2.12, *p* = 0.001) and arrival (*F*_38,188_  = 1.71, *p* = 0.013) differed among years, with the earliest migration occurring in 2009 (Appendix S2, Figure 2). Within 10 days of the average date of departure or arrival, absolute measures of daily weather also differed among years (Wilks' λ = 0.641, *F*_8,112_  = 3.47, *p* = 0.001). Canonical correlation analysis, however, indicated that overall significance was most influenced by mean snow depth (*F*_2,61_  = 5.55, *p* = 0.006; Appendix S2), which was greatest in 2008. Mean dates of departure were highly synchronous among three of four study areas (all *p*>0.10), with deer from North Ridge departing earliest (all *p*<0.02); dates of arrival were similar among study areas (*F*_38,188_  = 1.51, *p* = 0.212; Figure 3).

Within study areas, measures of tortuosity varied little among individuals (all *p*>0.05); therefore, we used the average value for each study area as the buffer width for each migration path, the least tortuous being South Magnolia (ANOVA, *F*_3,180_  = 4.68, *p* = 0.004; North Ridge  = 1.08±0.060, North Magnolia  = 1.06±0.052, Ryan Gulch  = 1.07±0.076, South Magnolia  = 1.03±0.034). Mean well-pad density along migration routes (pads/km^2^) was lowest in North Ridge and North Magnolia, becoming progressively higher through Ryan Gulch and South Magnolia (ANOVA, *F*_3,188_  = 45.93, *p*<0.001; North Ridge <0.01±0.111, North Magnolia  = 0.05±0.014, Ryan Gulch  = 0.18±0.107, South Magnolia  = 0.19±0.132). Similarly, rates of movement by females during migration differed among study areas (*F*_3,188_  = 6.42, *p*<0.001), and increased with well-pad density. Movement rates (km/day) were highest for deer migrating through the most developed study site (South Magnolia  = 11.6±6.38) compared with deer in the least developed areas (North Ridge  = 7.6±4.33, North Magnolia  = 7.5±3.55). Additionally, distance traveled differed among study areas (*F*_3,188_  = 6.30, *p*<0.001), with deer from North Ridge and North Magnolia traveling farther than deer from Ryan Gulch and South Magnolia (North Ridge  = 53±31.8 km, North Magnolia  = 47±20.7 km, Ryan Gulch  = 38±17.0 km, South Magnolia  = 36±10.2 km). Elevations of migratory paths varied among study areas (ANOVA, *F*_3,188_  = 16.57, *p*<0.001; North Ridge  = 2,316±22.6 m, North Magnolia  = 2,417±31.2 m, Ryan Gulch  = 2,468±12.9 m, South Magnolia  = 2,497±15.2 m), but remained relatively consistent within study areas among years (*F*_2,188_  = 2.81, *p* = 0.063; mean  = 2,420 m, SE  = 11.5).

Median estimated age of female deer was 3.5 and ranged from 1.5 to >10.5 years-of-age. Age of females migrating was similar among years (ANOVA, *F*_1,149_  = 0.02, *p* = 0.89) and study areas (*F*_3,149_  = 1.57, *p* = 0.19). Mean IFBFat was 6.8% (SE  = 0.12%) and ranged from 3.9 to 10.7%. Ingesta-free body fat of females was similar among years (*F*_1,149_  = 0.09, *p* = 0.76) and study areas (*F*_3,149_  = 2.06, *p* = 0.11).

### Predictors of Spring Migration

#### Extrinsic Variables

**Figure 4:**
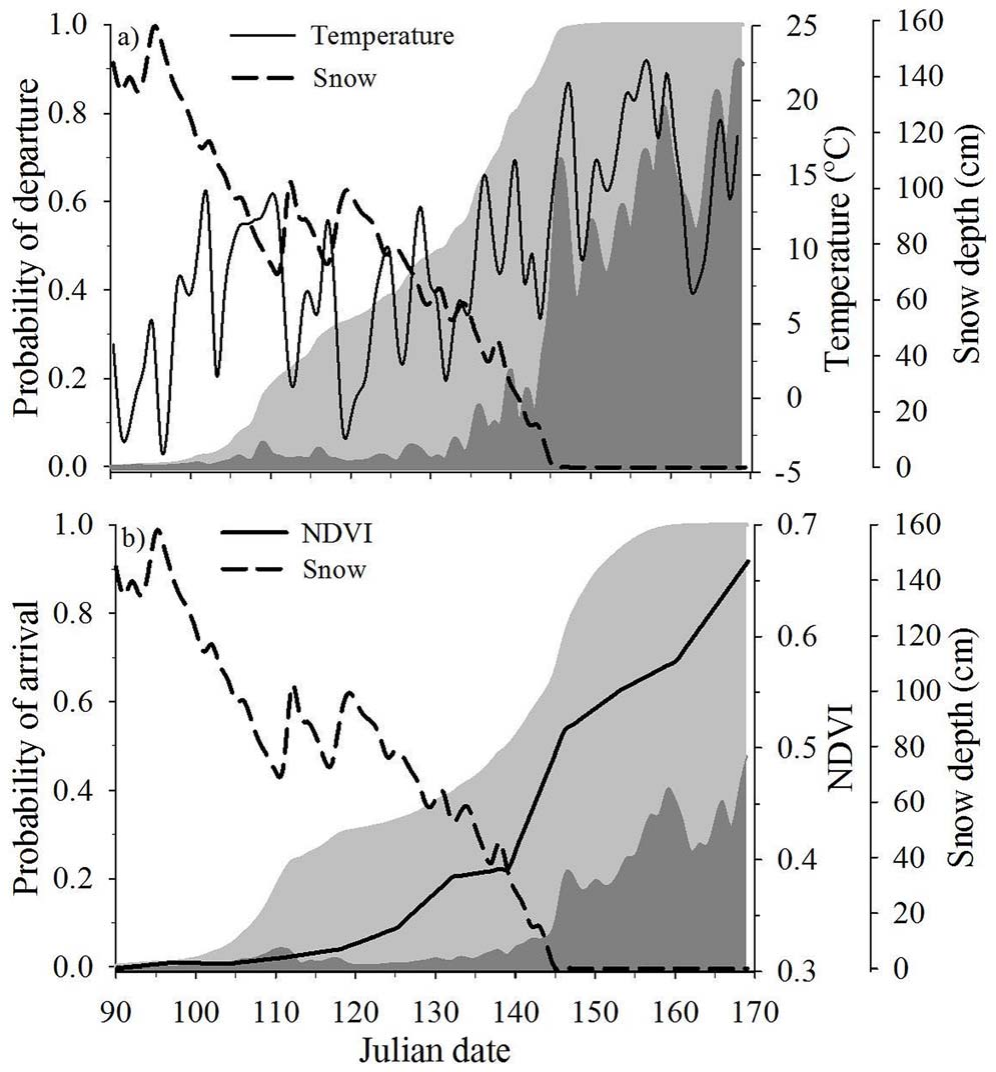
Probability of spring migration relative to environmental variables. Model-averaged estimates of the daily probability of migration (dark grey shaded region) and cumulative proportion migrated (light grey shaded region), average daily temperature (°C), daily snow depth (cm), and normalized difference vegetation index (NDVI) relative to Julian date of departure from winter range (a) and arrival on summer range (b) during spring migration of adult female mule deer, Piceance Basin, Colorado, USA, 2010.

**Figure 5:**
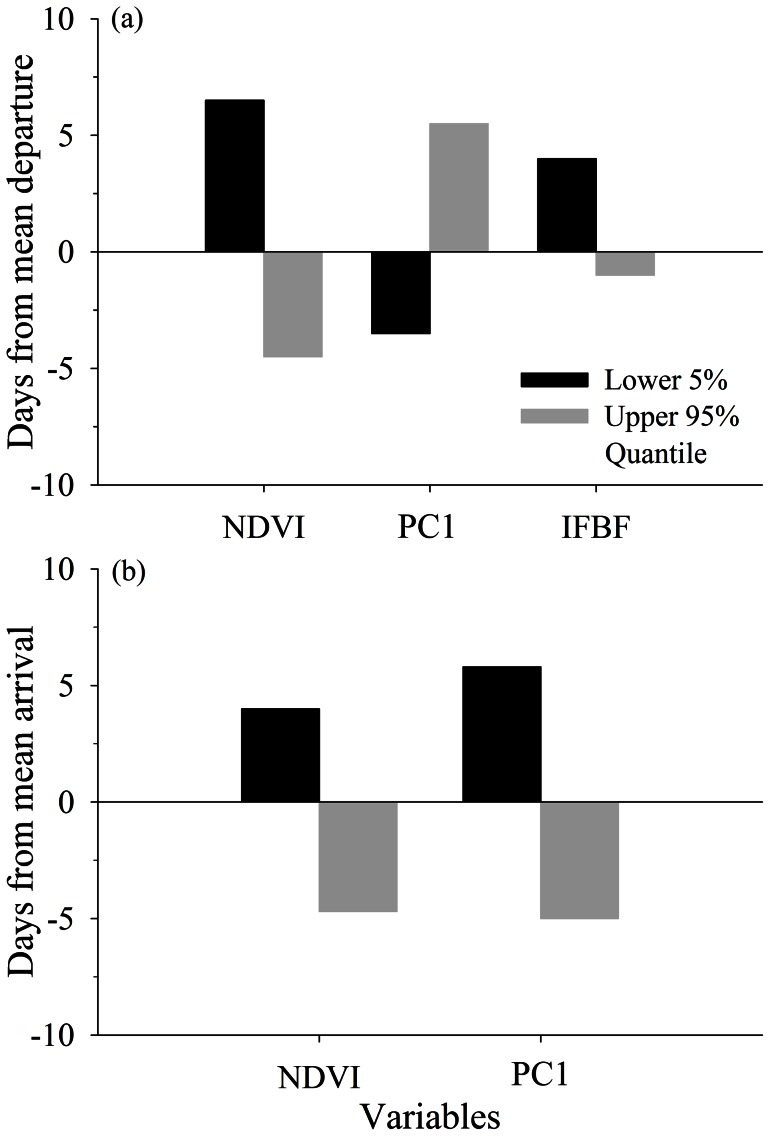
Predicted effect of migration under varying conditions. Relative difference in days of mean departure and arrival of adult female mule deer for variables identified to influence spring migration in the Piceance Basin Piceance Basin, Colorado, USA, during 2010. Estimates illustrate the predicted effects for the upper 95% and lower 5% quantiles of the normalized difference vegetation index (NDVI); distance traveled, rate of travel, well-pad density (PC1); and ingesta-free body fat (IFBFat), relative to the average date of migration. Predicted effects of each respective variable were determined by holding all other variables constant at their mean.

Departure from winter range was associated with daily absolute snow depth and temperature (WPC2), daily solar radiation (WPC4), daily change in snow depth (WPC5), and NDVI on winter range (WR-NDVI; importance weight >0.70; Table 1). Daily probability of leaving winter range was higher with reduced snow depth, warmer temperatures, cloudier days, and greater values of NDVI (Figs. 4, 5). Deer that wintered in areas with early increases in NDVI (95% quantile) left winter range 4.5 days prior to the mean departure day, whereas deer that experienced slower spring green-up (5% quantile) left winter range 6.5 days after mean departure day (Figure 5).

**Table 1:** Model-averaged parameter estimates, 90% CI, and Akaike importance weights for interval-censored models describing the relationship between the daily probability of departure from winter range for spring migration of mule deer, Piceance Basin, Colorado, USA, 2008–2010.

			90% CI			
Model Group	Parameter	Estimate	Lower	Upper	Importance Weight	Significant
Extrinsic	WPC1	−0.07	−0.17	0.03	0.52	No
	WPC2	−3.57	−4.11	−3.02	1.00	Yes
	WPC3	−0.06	−0.16	0.03	0.49	No
	WPC4	−0.64	−0.90	−0.37	1.00	Yes
	WPC5	−0.30	−0.49	−0.10	0.91	Yes
	WR-NDVI	5.49	2.67	8.31	1.00	Yes
	SR-NDVI	n/a	n/a	n/a	0.00	No
	SA	n/a	n/a	n/a	1.00	Yes
	Year	n/a	n/a	n/a	1.00	Yes
Intrinsic	PC1	−0.28	−0.44	−0.12	0.96	Yes
	PC2	0.01	−0.05	0.06	0.28	No
Life-history	IFBF	0.10	0.00	0.19	0.75	Yes
	Age	0.00	−0.02	0.01	0.25	No

Extrinsic variables included: change in temperature and humidity (WPC1), absolute snow depth and daily temperature (WPC2), precipitation (WPC3), solar radiation (WPC4), changes in snow depth (WPC5), normalized difference vegetation index on winter range (WR-NDVI), on summer range (SR-NDVI), and study area (SA). Intrinsic variables included: distance migrated, average daily distance traveled, well-pad density along migration routes (PC1), and average elevation on summer range (PC2). Individual covariates were ingesta-free body fat (IFBFat), age in years (Age). Year (Year) was included as a nuisance parameter.

Timing of arrival on summer range was affected by daily change in temperature (WPC1) and snow depth (WPC5), NDVI on summer range (SR-NDVI), and an interaction between absolute snow depth and temperature (WPC2) and NDVI of summer range (SR-NDVI; Table 2). Similar to departure from winter range, probability of arrival on summer range increased as snow depths declined, temperatures warmed, and NDVI increased (Figs. 4, 5). Although NDVI on summer range had a positive effect on the probability of arrival on summer range, that relationship was dampened by increases in snow depth (Table 2). Individuals arrived 4.7 days earlier than average when their summer ranges displayed high values of NDVI (95% quantile) compared with a 4-day delay from the mean for individuals with summer ranges with low NDVI levels (5% quantile; Figure 5).

**Table 2:** Model-averaged parameter estimates, 90% CI, and Akaike importance weights for interval-censored models describing the relationship between the daily probability of arrival to summer range for spring migration of mule deer, Piceance Basin, Colorado, USA, 2008–2010.

			90% CI			
Model Group	Parameter	Estimate	Upper	Lower	Importance Weight	Significant
Extrinsic	WPC1	−0.12	−0.23	−0.01	0.76	Yes
	WPC3	−0.06	−0.15	0.03	0.45	No
	WPC4	−0.10	−0.20	0.00	0.64	No
	WPC5	−0.60	−0.76	−0.44	1.00	Yes
	SR-NDVIxWPC2	−3.98	−4.67	−3.29	1.00	Yes
	SR-NDVI	2.16	0.68	3.64	1.00	Yes
	WR-NDVI	n/a	n/a	n/a	0.00	No
	SA	n/a	n/a	n/a	1.00	No
	Year	n/a	n/a	n/a	1.00	Yes
Intrinsic	PC1	0.57	0.42	0.71	1.00	Yes
	PC2	−0.09	−0.19	0.01	0.64	No
Life-history	Age	0.00	−0.02	0.02	0.34	No
	IFBF	−0.02	−0.08	0.05	0.63	No

Extrinsic variables included: change in temperature and humidity (WPC1), absolute snow depth and daily temperature (WPC2), precipitation (WPC3), solar radiation (WPC4), changes in snow depth (WPC5), normalized difference vegetation index on winter range (WR-NDVI), on summer range (SR-NDVI), interaction between SR-NDVI and WPC2 (SR-NDVIxWPC2), and study area (SA). Intrinsic variables included: distance migrated, average daily distance traveled, well-pad density along migration routes (PC1), and average elevation on summer range (PC2). Individual covariates were ingesta-free body fat (IFBFat), age in years (Age). Year (Year) was included as a nuisance parameter.

#### Intrinsic Variables

PC1, reflecting distance, rate of travel, and well-pad density, affected daily probability of departure from winter range (Table 1). Deer that traveled greater distances, at slower rates, through less natural-gas development (5% quantile) departed winter range 3.5 days earlier than average, whereas deer that traveled faster, over shorter distances, through greater levels of natural-gas development (95% quantile) remained on winter range 5.5 days longer than average (Figure 5). That same intrinsic variable (PC1) also affected timing of arrival on summer range; however, the relationship was the opposite of that for departure from winter range (Table 2). Deer that traveled slower for greater distances, through less development (5% quantile) arrived on summer range later than deer that traveled faster, over shorter distances, through greater development (95% quantile, Figure 5). PC1 had an effect similar in magnitude to that of NDVI on timing of arrival, with a deviation from the mean departure date of approximately 5.5 days (Figure 5). Elevation (PC2) of summer and winter ranges did not have a direct effect on probability of migration (Tables 1, 2). In a post-hoc analysis, however, we exchanged NDVI for elevation from the top model; elevation of summer range was significant (90% CI not overlapping 0) and positively related (β = 0.001) to timing of arrival on summer range.

#### Life-history Characteristics

Only IFBFat influenced probability of departure from winter range. Deer in better nutritional condition (95% quantile) were more likely to initiate spring migration earlier than deer with low body fat (5% quantile), which departed winter range 5 days later than deer in the upper 95% quantile (Figure 5). Timing of departure differed significantly among study areas, as did year for departure and arrival models (Tables 1, 2), indicating that some other factors among study areas and years still remained unexplained by our predictor variables.

## Discussion

Migration by large herbivores is thought to be a strategy aimed at enhancing fitness, by increasing access to food, escape from predators, and avoidance of risky environmental conditions [8], [14]. We evaluated whether anthropogenic disturbance modified patterns of spring migration of mule deer while accounting for other factors known to underpin migration of large herbivores. Initiation of spring migration was linked closely to patterns of local weather and plant phenology. Migration was delayed with increased winter severity associated with decreased temperature, increased humidity, and increased snow depth. Deep snow along migratory routes hampered travel of migrating deer by delaying their arrival to summer range, even if green-up was occurring at their destination. Indeed, migration strategies of ungulates are plastic and individuals delay migration in years with heavy snow pack and late green-up in spring, and migrate early in years with low snow pack and early emergence of vegetation [3], [12], [17]. Although weather and plant phenology helped synchronize spring migration, anthropogenic disturbance affected how individuals responded to such external stimuli.

Female mule deer departed winter range and arrived on summer range following patterns of green-up (NDVI) on each respective range. Patterns of vegetation green-up on winter range affected timing of departure, whereas arrival on summer range was related to green-up on those areas. The ability of ungulates to follow the “green wave” along spatial and elevational gradients is a well-documented phenomenon, and is a critical aspect of their behavioral ecology, which allows individuals to enhance nutritional gain via access to high-quality forage during a crucial time of year [12], [17], [36]. Delayed migration to high-elevation ranges was more accurately described by patterns of NDVI. Indeed, when we exchanged NDVI for elevation from the top model, elevation of summer range was negatively related to timing of arrival, which supports the hypothesis that mule deer are following emerging vegetation along spatial and elevational gradients.

Garrott et al. [3] hypothesized that deer must first improve their physiological condition prior to incurring the energetic costs associated with spring migration. In our study, female mule deer in better nutritional condition departed winter range earlier than females with lower ingesta-free body fat (IFBFat). Locomotive costs associated with migration are much less costly for large ungulates [37] compared with avian taxa, which may deplete substantial somatic reserves during migration [38]. Rather, we hypothesize that female mule deer with depleted nutritional reserves exhibited risk-averse behavior by remaining on winter range longer, where forage resources were likely less palatable and diverse, but more predictable (sensu [12]). Moreover, females in better nutritional condition that left winter range earlier risked encountering deep snow or late-winter storms at higher elevations, but could have been rewarded with newly emergent vegetation, thereby exhibiting risk-prone behavior [12]. Consequently, individual migration decisions can result in fitness inequalities, which may influence population dynamics [39].

Characteristics of migration routes of individuals affected how well their migration was synchronized with changes in weather and plant phenology; well-pad density and distance traveled likely affected timing of migration indirectly by influencing rate of travel during migration. Deer from the least-developed areas traveled slower over greater distances compared with deer that migrated through more developed areas over shorter distances – an effect that was comparable to green-up on timing of migration (Figure 5). As reported by Lendrum et al. [15], mule deer migrating through the study area of greatest well-pad density had longer step lengths compared with deer migrating through the least-developed areas. This outcome is consistent with the observations noted herein; that mule deer traveling through areas of high development traveled faster, and as a result, departed winter range later but still arrived to summer range earlier compared with deer migration through areas of lesser development. Unfortunately, we were not able to disentangle rate of travel and well-pad density from distance traveled, and had no pre-development data, which might have further clarified this outcome. Nevertheless, ungulates commonly avoid areas of human disturbance, including roads and well pads, especially in late winter and at parturition [19], [20], [40], although well pads were not avoided by mule deer during this study [15]. We hypothesize that instead of avoiding disturbed areas during migration, mule deer altered timing and rate of movement to reduce exposure to disturbance, potentially reducing net energetic gain [37]. This display of behavioral plasticity may be an important strategy where mule deer use traditional routes during migration that are affected by anthropogenic disturbances.

To compensate for the increased rates of travel in areas of high disturbance, female deer that migrated faster departed winter range later, but still arrived on summer range earlier than deer that traveled through less-developed areas. This alteration in timing could disconnect timing of migration from phenological progression [41]. For migratory ungulates, failure to time life-history events in accordance with advances in plant phenology can have adverse effects on fitness by reducing net energetic gains [42]. Herbivores track phenological progression of forage plants by moving across landscapes to acquire newly emergent vegetation [30], a strategy of particular importance to pregnant females supporting the increasing demands of late gestation [17]. The relatively minor shift in departure and arrival dates as a function of disturbance levels that we observed (∼8 days) may not contribute to a long term fitness consequence, but could do so if increased development activity intensified behavioral shifts in migratory patterns of mule deer [18]. Additionally, this potential disconnect between timing of migration and phenological progression could be exacerbated for deer that migrate greater distances or longer durations than those observed in the Piceance Basin.

Migration is a critical life-history characteristic of ungulates that is at risk of disruption because of habitat loss and fragmentation, largely resulting from anthropogenic disturbances [4], [6]. In some situations, the advantages acquired by migration could be outweighed by the risk, additional time, and energetic costs associated with avoidance of increased human development [5], [7], [18]. Despite high levels of energy development in the Piceance Basin, local weather patterns and plant phenology remained the predominant factors driving patterns of migration in mule deer; however, exposure to disturbance altered how individual deer responded to those environmental factors. As the level of natural-gas development expands across the Intermountain West, large areas of habitat for mule deer are being rapidly converted into gas fields consisting of networks of access roads, well pads, pipelines, and other infrastructure, which have potential to alter migratory behavior [18], [20]. Mule deer in the Piceance Basin appear to avoid negative effects from development activity through behavioral shifts in timing and rate of migration. Continued monitoring of mule deer and energy-development interactions are necessary to identify potential development strategies that minimize behavioral shifts in traditional migratory patterns.

## Supporting Information

Appendix S1 (DOC)

Appendix S2 (DOC)
