# Supplementary material for: Migrating Mule Deer: Effects of Anthropogenically Altered Landscapes
Source: PLoS One. 2013 May 14;8(5):e64548. doi: 10.1371/journal.pone.0064548 (PMC3653929; doi:10.1371/journal.pone.0064548)
Supplement: Appendix S1 — (DOC) [file pone.0064548.s001.doc]

**Appendix I.**  Component loadings for principal component analysis for extrinsic and intrinsic variables used to model timing of spring migration for mule deer in the Piceance Basin, Colorado, USA, 2008–2010.

| Variables | Component loadings | | | | |
| --- | --- | --- | --- | --- | --- |
| Extrinsic | 1 | 2 | 3 | 4 | 5 |
| Snow depth | 51.49 | 2.33 | -1.88 | 0.37 | 0.19 |
| ∆ Snow | 6.60 | -1.86 | 14.89 | 0.75 | -0.19 |
| Temperature | -4.00 | 3.79 | 0.27 | 3.29 | 3.19 |
| ∆ Temperature | -0.32 | 3.64 | 0.26 | 0.23 | 2.04 |
| Humidity | 3.40 | -20.96 | 0.41 | -4.62 | 1.60 |
| ∆ Humidity | 0.95 | -20.97 | -1.81 | 5.22 | -0.68 |
| Solar radiation | -0.51 | 1.38 | 0.02 | 0.34 | -0.15 |
| ∆ Solar | 0.11 | 0.32 | 0.12 | -0.02 | -0.11 |
| ∆ Precipitation | 0.07 | -1.33 | -0.23 | 0.38 | 0.21 |
| Precipitation | -0.19 | -1.32 | -0.17 | 0.08 | 0.45 |
| Intrinsic |  |  |  |  |  |
| Distance | -0.81 | 0.14 |  |  |  |
| Rate | 0.75 | -0.20 |  |  |  |
| Well density | 0.73 | 0.33 |  |  |  |
| Elevation | 0.03 | 0.95 |  |  |  |
