# Supplementary material for: Migrating Mule Deer: Effects of Anthropogenically Altered Landscapes
Source: PLoS One. 2013 May 14;8(5):e64548. doi: 10.1371/journal.pone.0064548 (PMC3653929; doi:10.1371/journal.pone.0064548)
Supplement: Appendix S2 — (DOC) [file pone.0064548.s002.doc]

**Appendix II.** Mean date of departure and arrival, and weather variables for 10 days prior to and post mean departure and arrival dates of spring migration of mule deer, Piceance Basin, Colorado, USA, 2008–2010. Snow depth was collected from SNOTEL weather station; temperature, humidity, and solar radiation, was collected from Western Regional Climate Center.

| Year | Day | | NDVI | | Snow Depth (cm) | | Temperature (°C) | | Humidity (%) | | Solar Radiation (Watts/m2) | |
| --- | --- | --- | --- | --- | --- | --- | --- | --- | --- | --- | --- | --- |
| Departure | Mean | SD | Mean | SE | Mean | SE | Mean | SE | Mean | SE | Mean | SE |
| 2008 | 134.30 | 10.62 | 0.34 | 0.001 | 77.05 | 3.91 | 10.11 | 1.12 | 50.24 | 4.27 | 6.69 | 0.41 |
| 2009 | 123.90 | 5.84 | 0.31 | 0.001 | 57.69 | 5.13 | 10.37 | 0.82 | 41.57 | 4.28 | 7.26 | 0.43 |
| 2010 | 132.50 | 7.75 | 0.34 | 0.001 | 61.21 | 4.02 | 8.38 | 0.87 | 50.70 | 4.11 | 6.88 | 0.40 |
| Arrival |  |  |  |  |  |  |  |  |  |  |  |  |
| 2008 | 142.40 | 9.81 | 0.42 | 0.002 | 59.75 | 5.55 | 11.25 | 1.11 | 45.86 | 4.41 | 7.16 | 0.46 |
| 2009 | 131.10 | 6.16 | 0.38 | 0.002 | 20.68 | 4.79 | 12.67 | 0.81 | 38.00 | 3.44 | 7.51 | 0.42 |
| 2010 | 139.50 | 7.72 | 0.40 | 0.001 | 37.62 | 5.19 | 10.35 | 1.07 | 48.33 | 4.47 | 6.86 | 0.39 |
